# Correlation between decreased PaCO_2_ and thrombus site and 90d prognosis in patients with acute pulmonary embolism

**DOI:** 10.3389/fmed.2025.1601169

**Published:** 2025-06-17

**Authors:** Danrong Yang, Yi Liu

**Affiliations:** Department of Respiratory and Critical Care Medicine, Shanghai Sixth People's Hospital Affiliated to Jiao Tong University School of Medicine, Shanghai, China

**Keywords:** pulmonary embolism, PaCO_2_, prognosis, relevant factors, D-dimer

## Abstract

**Objective:**

This study aimed to investigate the correlation between decreased partial pressure of arterial carbon dioxide (PaCO_2_) and thrombus location, as well as its association with 90-day prognosis in patients with acute pulmonary embolism (PE).

**Method:**

A retrospective analysis was conducted on the risk factors, clinical characteristics, and diagnostic findings of PE patients presenting with decreased PaCO_2_ at our hospital.

**Results:**

Multivariate logistic regression analysis revealed that decreased PaCO_2_ in PE was significantly associated with thrombosis in the right pulmonary artery, thrombosis in the left lower lobe pulmonary arteries, and pulmonary hypertension (PH). Pearson correlation analysis showed a weak negative correlation between decreased PaCO_2_ and PASP and PVOI. Spearman correlation analysis indicated a weak negative correlation between decreased PaCO_2_ and computed tomography severity (CTS)-defined risk PE. Kaplan–Meier survival analysis demonstrated that decreased PaCO_2_ was not associated with 90-day mortality (χ^2^ = 0.263, *p* = 0.608).

**Conclusion:**

A significant proportion of PE patients presented with decreased PaCO_2_, which was correlated with thrombosis in the right pulmonary artery and left lower lobe pulmonary arteries. However, decreased PaCO_2_ was not associated with 90-day mortality in PE patients.

## 1 Introduction

Arterial partial pressure of carbon dioxide (PaCO_2_) is a common parameter in blood gas analysis, reflecting lung ventilation function. Hyperventilation leads to a decrease in PaCO_2_, while insufficient ventilation results in increased PaCO_2_. In pulmonary embolism (PE) patients, decreased PaCO_2_ is often observed and is currently attributed to pulmonary artery blockage by thrombi, which reduces alveolar surfactant, lung compliance, and alveolar diffusion capacity. This disrupts the ventilation/perfusion (V/Q) ratio, leading to pulmonary arteriovenous shunting, local atelectasis, pleural effusion, hypoxemia, and compensatory hyperventilation (resulting in decreased PaCO_2_) (1, 2). However, we observed that some PE patients exhibited decreased PaCO_2_, while others did not, even in cases with smaller thrombi. This suggests that other factors (e.g., nerve distribution in the lungs) may contribute to decreased PaCO_2_. Pulmonary vessels have sympathetic and vagus nerve distribution. When the thrombus stimulates the pulmonary vessel wall, the sympathetic nerve is excited, which leads to vasoconstriction and increased pulmonary vascular pressure. Nevertheless, whether the stimulation of pulmonary nerves by thrombus leads to decreased PaCO_2_, and the relationship between thrombus location and decreased PaCO_2_ in PE patients is limited research.

This study retrospectively analyzed data from PE patients with decreased PaCO_2_ to explore the causes of decreased PaCO_2_ and its correlation with thrombus location and 90-day prognosis.

## 2 Methods

### 2.1 Subjects

A total of 801 PE patients (418 males and 383 females, aged 22–94 years) diagnosed via computed tomography pulmonary angiography (CTPA) at the Department of Respiratory and Critical Care Medicine of Shanghai Sixth People’s Hospital between September 2018 and April 2024 were included. Arterial blood gas analysis (with nasal catheter oxygen inhalation in some patients) was performed within 4 h of PE diagnosis. Patients were stratified into two groups based on PaCO_2_ levels: a decreased group (< 35 mmHg, 332 cases) and a non-decreased group (≥ 35 mmHg, 469 cases).

The inclusion criteria followed the diagnostic guidelines for pulmonary thromboembolism published by the Chinese Thoracic Society (CTS) in 2018 (3). Exclusion criteria included: patients diagnosed with CTPA at other hospitals who sought further treatment at our hospital; patients with incomplete medical records or missing critical research data; patients unable to undergo CTPA due to factors such as obesity, severe renal insufficiency, or contrast allergy; patients who underwent pulmonary perfusion/ventilation imaging or other diagnostic methods; patients with severe cardiomyopathy, valvular heart disease, acute coronary syndrome, acute infection, acute cerebrovascular disease, or chronic pulmonary thromboembolism; and patients without arterial blood gas analysis within 4 h of PE diagnosis.

### 2.2 Treatment methods

Following PE diagnosis, anticoagulant therapy was initiated. Low-molecular-weight heparin (100 IU/kg every 12 h) was administered subcutaneously to 369 patients. Warfarin (2.5 mg) was introduced orally 24–48 h after the start of low-molecular-weight heparin. Both low-molecular-weight heparin and warfarin were used concurrently for 4–5 days. Once prothrombin time (PT) and international normalized ratio (INR) reached 2.0–3.0 for two consecutive days, low-molecular-weight heparin was discontinued, but warfarin was continued orally. When INR was less than 2.0, warfarin doses was increased 0.625 mg. When INR was more than 3.0, warfarin doses should be reduced or even discontinued. In 432 patients, rivaroxaban was used (15 mg orally twice daily for 3 weeks, followed by 20 mg once daily for 9 weeks).

This study was approved by the Ethics Committee of Shanghai Sixth People’s Hospital, Shanghai Jiao Tong University [No.: 2023-KY-138(K)]. All procedures adhered to the ethical standards of the institutional and national research committees and the principles of the 1964 Helsinki Declaration and its amendments. Informed consent was waived due to the retrospective design.

### 2.3 Data collection

The following data were retrospectively collected and analyzed: gender, age, body mass index (BMI), smoking history, underlying diseases (hypertension, coronary heart disease, stroke, diabetes, recent fractures within the past 4 weeks, recent fixation or surgery, malignancy, chronic pulmonary disease), major symptoms (dyspnea, chest pain, shortness of breath, etc.), arterial blood gas analysis, plasma D-dimer (D-D), N-terminal pro-Brain Natriuretic Peptide (NT-proBNP), cardiac troponin I (cTNI), electrocardiogram findings, pulmonary artery systolic pressure (PASP; measured via echocardiography), ultrasound findings of lower extremity veins, thrombus location, pulmonary hypertension (PH; PASP > 40 mmHg), pulmonary vascular obstruction index (PVOI) (4), CTS-defined risk PE (low risk, intermediate-low risk, intermediate-high risk, high risk), simplified Pulmonary Embolism Severity Index (sPESI; scores 0, 1, 2, ≥3), treatment modalities, and outcomes.

### 2.4 Statistical analysis

Statistical analysis was performed using SPSS version 17.0. The Mann–Whitney U test was used for intergroup comparisons. Normally distributed data were expressed as mean ± standard deviation (Mean ± SD) and compared using the t-test. Categorical data were presented as frequencies (%) and compared using the χ^2^ test. A logistic regression model was constructed with decreased PaCO_2_ (+)/(−) as the dependent variable and gender, age, risk factors, underlying diseases, arterial blood gas parameters, D-dimer, cTNI, NT-proBNP, and thrombus location as independent variables to identify predictors of decreased PaCO_2_. Pearson correlation was used to assess relationships between PaCO_2_, PASP, and PVOI. Spearman’s correlation was used to analyze associations between PaCO_2_ and CTS-defined risk PE or sPESI scores. Kaplan–Meier survival analysis was performed to evaluate the correlation between decreased PaCO_2_ and 90-day mortality. A *p*-value < 0.05 was considered statistically significant.

## 3 Result

### 3.1 Basic information

The proportion of PE patients presenting with decreased PaCO_2_ was 41.39% (332/801). The mean age of PE patients in the decreased group was 66.2 ± 14.82 years, while in the non-decreased group, it was 66.88 ± 13.59 years. In the decreased group, 50.6% (168/164) were males, compared to 53.3% (250/219) in the non-decreased group. No statistically significant differences were observed in basic information between the decreased and non-decreased groups (*p* > 0.05). Additionally, no significant differences were noted in underlying diseases, symptoms, arterial blood gas parameters, NT-proBNP levels, time of onset, or elevated cTNI levels between the two groups (*p* > 0.05). Detailed results are presented in Tables 1, 2.

**Table 1:** Comparison of basic information (mean ± SD) (*n*, %).

General information	Decreased PaCO_2_ group (*n* = 332 cases)	Non-decreased group (*n* = 469 cases)	F/χ^2^ value	*p*-value
Male/female (cases)	168/164	250/219	1.181	0.277
Age (years)	66.2 ± 14.82	66.88 ± 13.59	3.06	0.810
BMI (kg/m^2^)	23.01 ± 3.35	23.08 ± 3.13	2.086	0.149
Smoking history (cases)	90 (27.1%)	133 (28.35%)	0.265	0.607
Symptom duration (days)	5.59 ± 4.48	5.70 ± 4.59	3.241	0.072
Oral contraceptive use (cases)	0	0	–	–
Long-haul travel history (cases)	0	0	–	–
Atrial fibrillation (cases)	0	0	–	–
Hypertension (cases)	96 (28.9%)	138 (29.4%)	0.097	0.755
Coronary heart disease (cases)	81 (24.3%)	125 (26.65%)	2.961	0.086
Diabetes mellitus (cases)	42 (12.6%)	52 (11.08%)	1.821	0.178
Malignant tumor (cases)	44 (13.2%)	66 (14.07%)	0.030	0.862
Surgery or trauma (cases)	35 (10.5%)	42 (8.95%)	2.916	0.088
Stroke history (cases)	31 (9.33%)	39 (8.31%)	1.013	0.315
Chronic pulmonary disease (cases)	85 (25.6%)	112 (23.8%)	1.227	0.268
Symptoms				
Cough	70 (21.08%)	99 (21.1%)	0.001	0.987
Fever	37 (11.14%)	49 (10.4%)	0.392	0.531
Chest pain	52 (15.66%)	77 (16.4%)	0.329	0.567
Syncope	25 (7.53%)	43 (9.16%)	2.708	0.100
Chest distress	109 (32.8%)	142 (30.2%)	2.852	0.092
SBP at admission (mmHg)	130.45 ± 13.43	129.97 ± 15.16	3.46	0.063
DBP at admission (mmHg)	78.43 ± 10.97	78.86 ± 10.51	1.685	0.195
Heart rate at admission (bpm)	89.05 ± 19.47	86.59 ± 18.63	3.376	0.067

*p < 0.05. BMI, body mass index; SBP, Systolic blood pressure; DBP, Diastolic blood pressure; bpm, Beats per min.

**Table 2:** Comparison of laboratory examination (mean ± SD) (*n*, %).

General information	Decreased group(n = 332 cases)	Non-decreased group(n = 469 cases)	F/χ^2^ values	*p*-values
sPESI Class (cases)			3.694	0.296
0	79 (23.79%)	131 (27.9%)		
1	127 (38.2%)	177 (37.73%)		
2	86 (25.9%)	121 (25.79%)		
≥ 3	40 (12.04%)	40 (8.52%)		
cTNI (ug/L)	0.106 ± 0.199	0.116 ± 0.410	1.792	0.181
Pro-BNP (ng/L)	922.89 ± 1143.12	754.79 ± 1009.58	0.864	0.353
PaO_2_ (mmHg)	77.22 ± 17.44	82.25 ± 19.91	6.73	<0.001*
PaCO_2_ (mmHg)	31.58 ± 2.86	41.13 ± 4.82	42.25	<0.001*
D-Dimer (mg/L)	6.72 ± 5.44	6.06 ± 5.70	1.419	0.223
PASP (mmHg)	34.24 ± 18.64	29.84 ± 9.06	17.966	<0.001*
PVOI (%)	29.65 ± 15.90	27.90 ± 15.95	0.087	0.768
Thrombus site (cases)			74.018	<0.001*
Main pulmonary artery	62 (18.6%)	56 (11.9%)		
Left pulmonary artery	141 (42.4%)	148 (31.5%)		
Right pulmonary artery	167 (50.3%)	168 (35.8%)		
Left superior pulmonary artery	136 (40.9%)	167 (35.6%)		
Left inferior pulmonary artery	209 (62.9%)	120 (25.5%)		
Right superior pulmonary artery	146 (43.9%)	205 (43.7%)		
Right middle lobe pulmonary artery	134 (40.3%)	196 (41.7%)		
Right inferior pulmonary artery	157 (47.2%)	291 (62.04%)		
CTS-defined risk PE			12.922	0.002*
Low-risk patients	103 (31.02%)	189 (40.2%)		
Intermediate-low-risk patients	133 (40.06%)	191 (40.7%)		
Intermediate-high-risk patients	96 (28.9%)	89 (18.9%)		
DVT (cases)	71 (21.38%)	101 (21.53%)	0.01	0.919
PH (cases)	100 (30.21%)	75 (15.9%)	89.602	<0.001*
Pleural effusion (cases)	63 (18.9%)	78 (16.63%)	3.485	0.062
Minor bleeding (cases)	10 (3.01%)	11 (2.34%)	1.35	0.246
Hospitalization duration (days)	9.8 ± 3.28	9.03 ± 3.13	1.141	0.286
90-day mortality (cases)	9 (2.71%)	10 (2.13%)	1.717	0.424

*p < 0.05. TNI, troponin I; BNP, B-type natriuretic peptide; PVOI, pulmonary vascular obstruction index; PaCO_2_, partial pressure of carbon dioxide; PaO_2_, oxygen partial pressure; DVT, deep vein thrombosis, PASP, Pulmonary artery systolic pressure; PH, Pulmonary hypertension; sPESI, simplified Pulmonary embolism severity index.

### 3.2 Univariate and multivariate logistic regressions

Univariate logistic regression analyses were conducted to evaluate the associations between clinical variables and decreased PaCO_2_ in PE patients. The results indicated that decreased PaCO_2_ was associated with intermediate-high-risk PE, pulmonary hypertension (PH), pulmonary artery systolic pressure (PASP), and thrombosis in the left or right pulmonary artery, as well as thrombosis in the left or right inferior pulmonary artery (*p* < 0.05).

Multivariate logistic regression analysis was performed for variables that showed statistically significant differences in the univariate analysis. It revealed that the risk of decreased PaCO_2_ in PE patients was linked to thrombosis in the right pulmonary artery (OR = 1.737, 95% CI: 1.093–2.760, *p* = 0.020), thrombosis in the left inferior pulmonary artery (OR = 2.447, 95% CI: 1.743–3.434, *p* < 0.001), and was positively correlated with PH (OR = 3.506, 95% CI: 1.263–9.726, *p* = 0.016). Additionally, the risk of decreased PaCO_2_ was linked to PaO_2_ (OR = 0.988, 95% CI: 0.98–0.997, *p* = 0.003), intermediate-high-risk PE (OR = 0.371, 95% CI: 0.148–0.926, *p* = 0.034), and thrombosis in the right inferior pulmonary artery (OR = 0.416, 95% CI: 0.297–0.582, *p* < 0.001), showing a negative correlation.

### 3.3 Pearson correlation analysis

**Figure 1:**
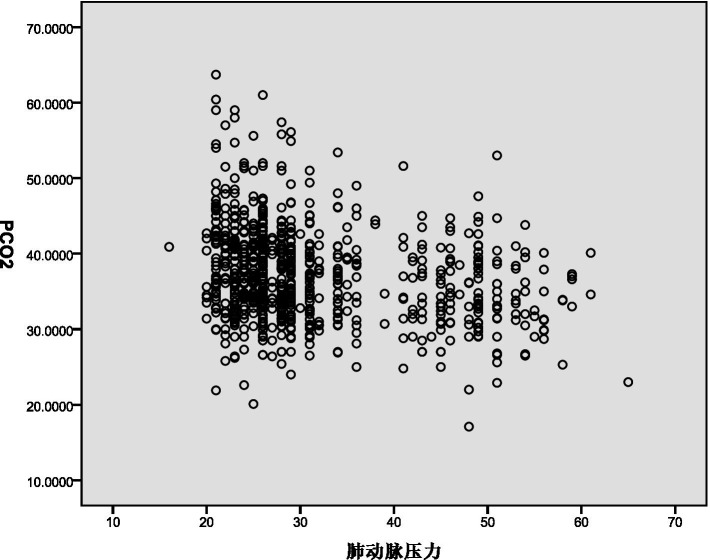
Pearson correlation coefficient assess the association between PaCO_2_ and PASP.

**Figure 2:**
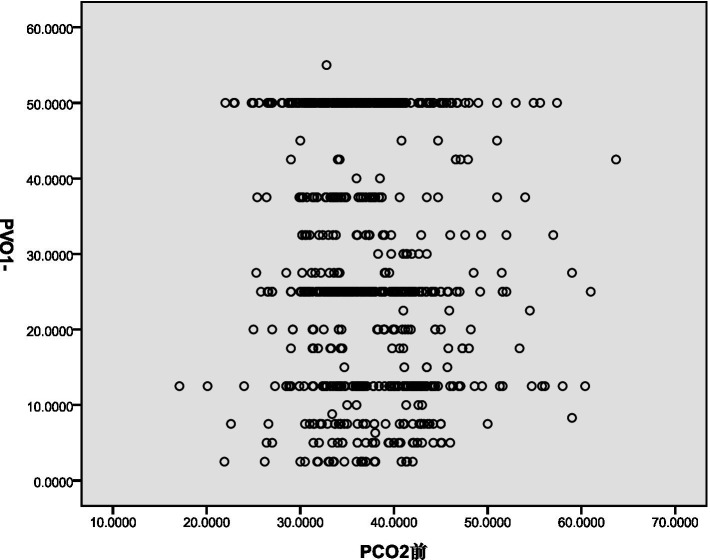
Pearson correlation coefficient assess the association between PaCO_2_ and PVOI.

The decrease in PaCO_2_ demonstrated a weak negative correlation with PASP (Rs = −0.248, *p* < 0.001) and pulmonary vascular obstruction index (PVOI) (Rs = −0.086, *p* = 0.015). These results are illustrated in Figures 1, 2.

### 3.4 Spearman correlation analysis

The decrease in PaCO_2_ demonstrated a weak negative correlation with CTS-defined risk PE (Rs = −0.124, *p* < 0.001). However, no correlation was observed between decreased PaCO_2_ and the simplified Pulmonary Embolism Severity Index (sPESI) (Rs = 0.041, *p* = 0.243).

### 3.5 Kaplan–Meier survival analysis

**Figure 3:**
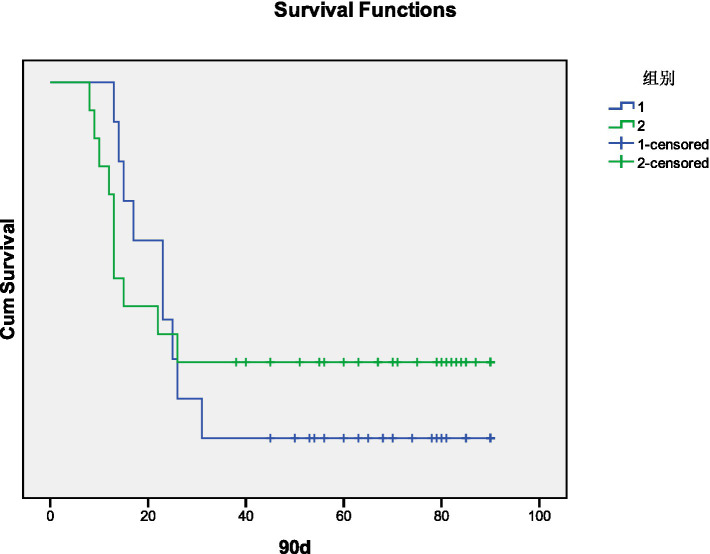
Kaplan–Meier curves for 90-day mortality after decreased PaCO_2_ in days (1. Decreased group, 2. Non-Decreased group).

Kaplan–Meier survival analysis indicated that decreased PaCO_2_ was not associated with 90-day mortality (χ^2^ = 0.263, *p* = 0.608). These results are shown in Figure 3.

## 4 Discussion

There are many factors affecting arterial partial pressure of carbon dioxide (PaCO_2_). Changes in PaCO_2_ may result from cardiopulmonary diseases or neurological and mental disorders. Chronic lung diseases such as chronic obstructive pulmonary disease (COPD) can lead to PaCO_2_ retention, while idiopathic pulmonary fibrosis (IPF) can cause a decrease in PaCO_2_. In this study, no statistically significant differences were observed in chronic lung diseases between the two groups. Additionally, vagus nerve (VN) excitation can increase PaCO_2_, whereas sympathetic nerve (SN) excitation can increase the respiratory rate and decrease PaCO_2_.

The lungs are innervated by both the sympathetic and parasympathetic nervous systems. The sympathetic nerves originate from the thoracolumbar segment of the spinal cord (T1–L2), emit postganglionic fibers after the sympathetic trunk, and form the anterior and posterior pulmonary plexuses with the vagus nerve. These nerves descend from the hilum of the lung along the trachea and pulmonary vessels, distributing around the bronchi and pulmonary vessel walls. Zhou et al. (5) found that the canine pulmonary sympathetic nerve bundle was primarily distributed in the posterior wall of the pulmonary trunk and the left pulmonary artery, with minimal distribution in the anterior wall of the pulmonary trunk and the right pulmonary artery. The longest and shortest distances between pulmonary artery nerve endings and the pulmonary artery lumen were 4,148 μm and 823 μm, respectively. Zhang et al. (6) reported that there were 2,160 sympathetic nerves around the pulmonary artery in dogs, with the largest concentration at the proximal end of the pulmonary artery bifurcation, accounting for 70 to 84% of the total. Most sympathetic nerves were distributed 1 to 3 mm below the intima at the beginning of the left and right pulmonary arteries and under the intima of the wall after the pulmonary artery bifurcation. Huang et al. (7) confirmed the presence of a large number of sympathetic nerves in the posterior wall of the main pulmonary trunk, the right wall, and the posterior wall of the pulmonary artery bifurcations. Wang et al. (8) found that the sympathetic nerve was overactivated in an animal model of pulmonary thrombosis, with significant increases in sympathetic neurotransmitters such as tyrosine hydroxylase and neuropeptide Y in lung tissue.

The results of this study showed that pulmonary thrombi were distributed in the right pulmonary arteries and/or the left lower pulmonary artery in the decreased PaCO_2_ group, consistent with the distribution of pulmonary sympathetic nerves. Multivariate logistic regression analysis identified thrombosis in the right pulmonary artery and left lower lobe pulmonary arteries as risk factors for decreased PaCO_2_. This may be due to the abundance of sympathetic nerves in the branches of the right and left pulmonary arteries, which are close to the vascular intima. Mechanical stimulation of the vascular wall by thrombi may activate para-pulmonary sympathetic nerves, leading to hyperventilation and decreased PaCO_2_. However, this study was a retrospective case analysis, and sympathetic neurotransmitters such as tyrosine hydroxylase, neuropeptide Y, and norepinephrine were not measured in PE patients with decreased PaCO_2_. Therefore, decreased PaCO_2_ cannot be considered a predictor of pulmonary vascular sympathetic nerve excitability.

Pearson correlation analysis revealed a weak negative correlation between PASP and decreased PaCO_2_. This may be due to the decrease in PaCO_2_ leading to a reduction in H^+^ concentration in the blood, stimulating chemoreceptors and causing reflex vasoconstriction, thereby increasing PASP (9, 10). Additionally, Pearson correlation analysis showed a negative correlation between the pulmonary vascular obstruction index (PVOI) and decreased PaCO_2_. Metafratzi et al. (11) demonstrated that a PaCO_2_ value of 30 mmHg or lower was associated with an arterial bed obstruction index of >50%. The greater the degree of thrombus blockage, the higher the pulmonary vascular pressure, and the more pronounced the vascular sympathetic nerve and mechanical stimulation, which may contribute to the decrease in PaCO_2_.

Spearman correlation analysis revealed a weak negative correlation between decreased PaCO_2_ and CTS-defined risk PE. However, no correlation was observed between decreased PaCO_2_ and the simplified Pulmonary Embolism Severity Index (sPESI). This may be because PASP and PVOI were higher in patients with decreased PaCO_2_ compared to the non-decreased group, indicating larger thrombus volume and greater pulmonary artery blockage, resulting in impaired cardiac function and higher risk stratification. However, as a retrospective study, systolic blood pressure and heart rate could not be recorded in real time, and some patients had received oxygen therapy at the time of diagnosis, which may explain the lack of correlation between decreased PaCO_2_ and sPESI scores. From these correlation studies, it is evident that decreased PaCO_2_ can indicate increased PVOI blockage, leading to elevated PASP and higher CTS-defined risk PE.

Kotsiou et al. (9) followed PE patients with decreased PaCO_2_ for 6 months and found that their 6-month survival rate was lower than that of patients with normal PaCO_2_. In our study, patients with decreased PaCO_2_ exhibited no hemodynamic instability, and the 90-day mortality in the decreased PaCO_2_ group was 9 cases, compared to 10 cases in the non-decreased PaCO_2_ group. Kaplan–Meier survival analysis indicated no correlation between decreased PaCO_2_ and 90-day mortality. The discrepancy with previous findings may be due to the shorter observation period in this study. Additionally, the 90-day mortality in both groups primarily involved patients with chronic end-stage pulmonary diseases, such as interstitial pulmonary disease combined with pulmonary hypertension, right heart failure, and chronic obstructive pulmonary disease with poor pulmonary function. These patients were bed-bound for extended periods, making them more susceptible to pulmonary thrombosis or infection, which likely contributed to the increased mortality.

However, this study has several limitations. First, its retrospective, single-center design limits the generalizability of the findings. To strengthen the results, further research with a larger, prospective, multicenter cohort, incorporating comprehensive medical histories and long-term follow-up data, is necessary. Second, because the number of patients in high-risk was small, and many patients could not be diagnosed by CTPA examination, so it was excluded from this study. Third, Many PE patients have COPD, or pneumonia, interstitial lung disease, pleural effusion, etc., which may have an impact on arterial blood gas, but it is difficult to the inability to accurately quantify the extent or scope of lung lesions prevented an analysis of their impact on PaCO_2_, so we did not include CT images in the manuscript.

## 5 Conclusion

The prevalence of decreased PaCO_2_ was elevated among PE patients. Decreased PaCO_2_ was associated with thrombosis in the right pulmonary artery and left lower lobe pulmonary arteries, consistent with the distribution of pulmonary sympathetic nerves. However, it remains unclear whether decreased PaCO_2_ can indicate increased sympathetic sensitivity. Decreased PaCO_2_ was negatively correlated with PVOI, PASP, and CTS-defined risk PE, indicating greater pulmonary vascular obstruction and higher risk stratification. However, decreased PaCO_2_ did not correlate with 90-day mortality.

## Data Availability

The raw data supporting the conclusions of this article will be made available by the authors, without undue reservation.
